# Alzheimer’s Disease-Like Neurodegeneration in *Porphyromonas gingivalis* Infected Neurons with Persistent Expression of Active Gingipains

**DOI:** 10.3233/JAD-200393

**Published:** 2020-06-15

**Authors:** Ursula Haditsch, Theresa Roth, Leo Rodriguez, Sandy Hancock, Thomas Cecere, Mai Nguyen, Shirin Arastu-Kapur, Sean Broce, Debasish Raha, Casey C. Lynch, Leslie J. Holsinger, Stephen S. Dominy, Florian Ermini

**Affiliations:** a Cortexyme, Inc., South San Francisco, CA, USA; b Laboratory for Neurotoxicity Studies, Virginia-Maryland College of Veterinary Medicine, Virginia Tech, Blacksburg, VA, USA

**Keywords:** Alzheimer’s disease, gingipain cysteine endopeptidases, *in vitro* techniques, multivesicular bodies, *Porphyromonas gingivalis*, synapses, tau protein

## Abstract

**Background:**

*Porphyromonas gingivalis* (*P. gingivalis*) and its gingipain virulence factors have been identified as pathogenic effectors in Alzheimer’s disease (AD). In a recent study we demonstrated the presence of gingipains in over 90% of postmortem AD brains, with gingipains localizing to the cytoplasm of neurons. However, infection of neurons by *P. gingivalis* has not been previously reported.

**Objective:**

To demonstrate intraneuronal *P. gingivalis* and gingipain expression *in vitro* after infecting neurons derived from human inducible pluripotent stem cells (iPSC) with *P. gingivalis* for 24, 48, and 72 h.

**Methods:**

Infection was characterized by transmission electron microscopy, confocal microscopy, and bacterial colony forming unit assays. Gingipain expression was monitored by immunofluorescence and RT-qPCR, and protease activity monitored with activity-based probes. Neurodegenerative endpoints were assessed by immunofluorescence, western blot, and ELISA.

**Results:**

Neurons survived the initial infection and showed time dependent, infection induced cell death. *P. gingivalis* was found free in the cytoplasm or in lysosomes. Infected neurons displayed an accumulation of autophagic vacuoles and multivesicular bodies. Tau protein was strongly degraded, and phosphorylation increased at T231. Over time, the density of presynaptic boutons was decreased.

**Conclusion:**

*P. gingivalis* can invade and persist in mature neurons. Infected neurons display signs of AD-like neuropathology including the accumulation of autophagic vacuoles and multivesicular bodies, cytoskeleton disruption, an increase in phospho-tau/tau ratio, and synapse loss. Infection of iPSC-derived mature neurons by *P. gingivalis* provides a novel model system to study the cellular mechanisms leading to AD and to investigate the potential of new therapeutic approaches.

## INTRODUCTION

Mounting evidence has identified *Porphyromonas gingivalis* (*P. gingivalis*) as a driving factor in the progression of Alzheimer’s disease (AD) [1, 2]. *P. gingivalis* specific cell free DNA can be detected in the cerebrospinal fluid and its protease virulence factors, arginine-gingipain (Rgp) and lysine-gingipain (Kgp), are present in the brains of over 90% of AD patients and correlate with tau and ubiquitin pathology [1]. *P. gingivalis* has been identified as a keystone pathogen for periodontitis [3, 4], which has been shown to be a risk factor for AD and a more rapid cognitive decline [5–10]. After oral infection in mice, *P. gingivalis* specific DNA and gingipains can be detected in the brain [1, 2, 11]. In one study using wild type mice, the major hallmarks of AD pathology, i.e., amyloid plaques, inflammation, microglial activation, hyperphosphorylated tau, and neuron loss, were reported after 22 weeks of infection [2]. To date, the mechanism of *P. gingivalis*-induced AD has not been elucidated, and it is imperative to develop a relevant *in vitro* model to advance our understanding of the underlying processes and to support the development of new therapies.

In our previous work, we demonstrated the susceptibility of tau protein to gingipain fragmentation [1], underlining the importance to study *P. gingivalis*-induced neurodegeneration in the context of functional tau protein. Tau is expressed in mature neurons only, and specific mechanisms that involve tau phosphorylation and aggregation have often been studied in transformed cell lines transfected with full-length tau protein [12]. Here, we use human inducible pluripotent stem cells (iPSC)-derived neuron cultures that express tau and display extensive neurite networks with functional synapses that allow the investigation of neuron-specific degenerative endpoints, such as hyperphosphorylation of tau or synapse loss [13].

*In vitro* cell infection studies with *P. gingivalis* have been conducted in a variety of cell types: gingival epithelial cells and fibroblasts, oral keratinocytes, endothelial cells from coronary arteries or umbilical cords, smooth muscle cells, hepatic cells, and dendritic cells [14–24]. The differences in attachment, invasion, persistence, and survival of *P. gingivalis* in the different culture systems are significant and highlight the need to better understand specific *P. gingivalis*-host interactions in highly specialized cells such as neurons.

*P. gingivalis* is an anaerobic, asaccharolytic, Gram-negative bacterium, and its survival in tissue depends heavily on its ability to invade and persist in host cells. The gingipains, Kgp and Rgp, are cysteine proteases attached on the bacterial surface or secreted into the environment. The gingipains are the major virulence factors that enable evasion of the cellular defenses from the host cell [25, 26]. For example, in coronary endothelial cells, gingipains have been shown to prevent the fusion of autophagosomes containing *P. gingivalis* with lysosomes, enabling a persistent residence of *P. gingivalis* in autophagosomes [24].

In this study, we describe an *in vitro* culture system to study *P. gingivalis*-host interactions of mature neurons and provide an initial characterization of AD-related neurodegeneration. We found that *P. gingivalis* can invade and survive in neurons and produce intraneuronal gingipains that are proteolytically active. After infection, 25% of neurons are lost in a time dependent manner. In surviving cells, full length tau was reduced with an increase in phosphorylation ratio over time, and this finding was associated with loss of neuronal synapses. At the ultrastructural level, intraneuronal *P. gingivalis* appeared free in cytoplasm or resided in lysosome-like structures, and infected neurons displayed accumulation of autophagic vacuoles and multivesicular bodies, a hallmark of dystrophic neurites in AD brains. Our results reveal that *P. gingivalis* can invade and survive inside neurons which leads to neuronal damage associated with AD.

## MATERIALS AND METHODS

### Aim and design

The aim of this study was to demonstrate that *P. gingivalis* can invade and persist in mature neurons. The result is an *in vitro* model system to study the neurodegenerative effects of *P. gingivalis* virulence on neurons. The study was designed to define the experimental parameters of neuron cultures and infection, to demonstrate viability of host and pathogen, and to provide an initial characterization of the time dependent effects on host and pathogen.

### NPC-derived neuronal cultures

Two neural precursor cell lines (NPCs XCL1 [#70901], XCL4[#70902]) were purchased from StemCell Technology, Inc. (Vancouver, Canada) and differentiated into neurons according to the manufacturer’s protocol (catalog numbers 08500 and 08510). Briefly, NPCs were plated onto Poly-l-Ornithine (PLO) and Laminin (MilliporeSigma, Burlington, MA) coated plates and cultured in STEMdiff™ Neuron Differentiation Media for 2 to 4 passages. Neuron maturation was achieved by changing from Neuron Differentiation to STEMdiff™ Neuron Maturation Media or Brainphys media (BrainPhys™ hPSC Neuron Kit, StemCell Technology) for 3 to 4 weeks. Neuronal maturation was confirmed in each culture by immunohistochemical staining of neuronal markers such as Tuj1 and Map2a. Cells were maintained at 5% oxygen and 5% carbon dioxide, and media changes were done every other day. All experiments were done in triplicates with 6 to 8 replicates in each experiment.

### Bacterial culture and inoculum preparation

*P. gingivalis*, ATCC 33277 (ATCC, Manassas, VA) was streaked onto Brucella Blood Agar with Hemin and Vitamin K plates (Hardy Diagnostics, Santa Maria, CA) and grown under anaerobic conditions at 37°C for 5–7 days. For infection, colonies were inoculated in tryptic soy broth (TSB; Hardy Diagnostics) with 0.5 mg/mL L-cysteine, 5*μ*g/mL hemin, and 2*μ*g/mL vitamin K and grown under anaerobic conditions at 37°C for 24–72 h. Cultures were then diluted 1 to 5 or 1 to 2.5 and grown under anaerobic conditions at 37°C for 16–24 h to OD600 0.5–0.8. Cultures were centrifuged at 3220 g room temperature (RT) for 20 min. Supernatant was discarded and bacterial pellet re-suspended and labeled with CellVue Claret Fluorescent Cell Linker Kit (MilliporeSigma). Dye was inactivated after a 30-s incubation with 5–10 mL 1 mg/mL bovine serum albumin (BSA; VWR, Radnor, PA). Bacteria was centrifuged at 3220 g RT for 20 min, supernatant discarded, and re-suspended in 1 mL 1X Dulbecco’s phosphate-buffered saline (PBS; ThermoFisher Scientific, Carlsbad, CA). Bacterial prep was mixed by pipette and an aliquot was taken for quantitative polymerase chain reaction (qPCR) analysis to determine the bacterial genome copy number per mL for the appropriate bacteria to cell ratio (BCR). The infectious bacterial titer was confirmed by colony forming unit (CFU) analysis.

### qPCR

Bacterial DNA was released by incubating samples at 95°C for 10 min. Primers and detection probes (MilliporeSigma) specific for the arginine-gingipain A (Rgp), lysine-gingipain (Kgp), and 16 s rRNA genes were used to detect and quantify *P. gingivalis* DNA. Primer sequences used: Rgp A fwd: 5’-GCTCTTTCCATAAACGTAAG-3’; Rgp A rev: 5’-GACACTCGTGAGATGAAG-3’; Kgp fwd: 5’-CGAAGCTGAAGTAGGAAC-3’; Kgp rev 5’-CAACCAAAGCCAAGAAGA-3’; 16 s fwd: 5’-ACGAGTATTGCATTGAATG-3’; 16 s rev: 5’-ACCCTTTAAACCCAATAAATC-3’. Detection probes used: Rgp A: FAM-TTCGGATCTTCGTTACGCATAATCA-BHQ-1; Kgp: Cyanine5-CACTAGCTGCCAATCCATCATT-BHQ-3; 16 s Hex-CGCTCGCATCCTCCGTATTAC-BHQ-1. Final concentrations of primers and probes were 0.5*μ*M and 0.15*μ*M, respectively. Samples were assayed in a 20*μ*L reaction using 2X Kapa Probe Fast qPCR Master Mix (Kapa Biosystems, USA). Cycling conditions were based on manufacturer instruction, enzyme activation for 3 min at 95°C followed by 40 PCR cycles (95°C for 3 s, 60°C for 10 s). The standard curve was prepared using 1:10 dilutions of a GBLOCK DNA sequence (IDT, Coralville, IA).

### Cell lysis and pooling for CFU assay

For CFU assay, cells were washed once with 1X PBS, then lysed with DI water for 30 min at 37°C. Lysate was collected, pooled by group, and kept on ice for analysis. Bacteria in lysate were quantified by CFU and qPCR analysis.

### RT-qPCR

Aliquots of cell lysate were added to two volumes of RNA Protect (Qiagen), RNA was extracted with a Lucigen MasterPure™ Complete DNA and RNA Purification Kit (Middleton, WI) according to manufacturer protocol. RNA was converted to cDNA by SuperScript™ IV First-Strand Synthesis System (ThermoFisher Scientific) according to manufacturer protocol. RT-qPCR was performed using the same conditions as described for qPCR. Primer and detection probes (IDT) specific for arginine gingipain B (RgpB), Kgp, and 16 s rRNA were used to quantify cDNA. Primer sequences used: RgpB fwd: 5’- CTTCCACTTTCACATCCTTTA-3’; RgpB rev: 5’- CTGACGATGGTGATATGG-3’; Kgp fwd: 5’- CAACCAAAGCCAAGAAGA-3’; Kgp rev: 5’- CGAAGCTGAAGTAGGAAC-3’; 16 s fwd: 5’- ACGAGTATTGCATTGAATG-3’; 16 s rev: 5’- ACCCTTTAAACCCAATAAATC-3’. Detection probes used RgpB: 5’-TEX615-CCTTATTGTATCCTACTACGGTGAGC-IAbRQSp-3’; Kgp: 5’-Cy5- CACTAGCTGCCAATCCATCATT-IAbRQSp-3’; 16s: 5’-Hex- AGAATAAGCATCGGCTAACTCCGT-IABkFQ-3’.

### Kgp and Rgp enzyme activity assays

Rgp enzymatic activity was measured using a kinetic colorimetric assay as previously described in Dominy et al. 2019 [12]. Samples were assayed in 100 mM Tris, 200 mM glycyl-glycine, 5 mM CaCl2, 10 mM Cys-HCl, pH 7.6 buffer with 1 mM *N*_*α*_-Benzoyl-L-arginine 4-nitroanilide hydrochloride (L-BAPNA, MilliporeSigma) as the substrate. Optical density (OD) 405 nm was read every 1.5 min for 1.5 h at 37°C. Purified Rgp (a kind gift from Barbara Potempa, University of Louisville) at a known concentration was used to generate the standard curve. Kgp enzymatic activity was measured using a kinetic fluorescent assay. Samples were assayed in 100 mM Tris, 75 mM NaCl, 2.5 mM CaCl2, 10 mM Cys-HCl buffer with 10*μ*M Z-His-Glu-Lys-MCA (MyBioSource, Inc., San Diego, CA) as the substrate. Assay was read at a wavelength of 460 nm on a Synergy H1 microplate reader (BioTek, Winooski, VT) every 1.5 min for 30 min at 37°C. Purified Kgp (a kind gift from Barbara Potempa, University of Louisville) at a known concentration was used to generate the standard curve.

### Kgp and Rgp activity probes

Fluorescent activity probes and methods associated with their use for irreversible binding of Kgp (COR553) or Rgp (COR619) were as described [1] with the below modifications. The detailed chemical synthesis and structure of compounds in the structural series for lysine gingipain inhibitors utilized to make these fluorescent probes is found in PCT/US2016/061197 and, referenced in application 62/459,456. Probe detection of active Kgp and Rgp was performed as described previously [1] Briefly, 1*μ*M COR553 or COR619 Cy5 probe, respectively was added into the medium of infected neuron cultures for 1 h at 37°C. Cells were washed in PBS and then lysed with DI water and centrifuged at 10000×g for 10 min at 4°C and resuspended in B-PER (ThermoFisher) at 10-fold concentration. The sample was prepared for blotting with Nupage LDS sample buffer (ThermoFisher) at 95°C and loaded onto denaturing AnyKd gel (Bio-Rad, Hercules, CA). After protein separation, the gel was imaged on ChemiDoc MP Imaging System (Bio-Rad) with Image Lab 6.01 software on the Cy5 channel.

### Western blots

Cells were lysed in RIPA buffer (MilliporeSigma) supplemented with proteinase (ThermoFisher) and phosphatase (PhosSTOP, Roche Diagnostics, Indianapolis, IN) inhibitors. The concentration of cell lysates was determined using bicinchoninic acid assay (BCA) (ThermoFisher). Samples were loaded onto 4–20% Mini-Protean TGX Precast Gels (Bio-Rad) and then transferred to PVDF membranes (Bio-Rad) using Trans-Blot Turbo System (Bio-Rad). Membranes were incubated with following primary antibodies: mouse anti-total tau A0024 (DAKO; 1:1000, # A0024), mouse anti-synapsin (ThermoFisher; 1:1000, MA531919), rabbit anti-Gapdh-HRP (Cell Signaling; 1:2000, #8884), rabbit anti-Rgp antibody CAB101 (1:1000) [1], and rabbit anti-Kgp antibody CAB102 (1:1000) [1]. All secondary antibodies were horseradish protein (HRP) conjugated (Vector; 1:5000 or 1:10000). Blots were imaged by chemiluminescent detection using Supersignal West Fento (ThermoFisher) and the ChemiDoc imaging system. Quantification was performed with Image Lab 6.0.1 software (Bio-Rad) by dividing the integrated OD of all bands of interest by the OD of GAPDH detected on the same blot.

### ELISA

Levels of phosphorylated tau and total tau in cell lysates were measured with the phospho-tau (Thr231)/total tau Kit from Meso Scale Diagnostics (Rockville, MD, K15121D) according to the manufacturer’s instructions. The cell lysates described above were diluted 50-fold in Tris Wash buffer containing 1% Blocker A (provided by the Meso Scale Diagnostic Phospho-Tau kit, Rockville, MD, K15121D) and incubated overnight in the Multi-spot 96 well 4-spot Phospho (Thr231)/total Tau plate shaking at 4°C. The next day, after washing and incubation with detection antibody according to the manufacturer’s instructions, phosphorylated tau and total tau were quantified with the MESO QuickPlex SQ 120 instrument (Meso Scale Diagnostics).

### Cell death assay

To determine cell death, the Image-iT^®^ DEAD Green™ viability stain (ThermoFisher, I10291) was added to the neuronal cultures at a final concentration of 100 nM and incubated for 1 h at 37°C. Cells where then washed with PBS and fresh media with NucBlue nuclear dye (NucBlue™ Live ReadyProbes™ Reagent, ThermoFisher, R37605) added before image acquisition. Dead cells were identified by the colocalization of DEAD Green dye with NucBlue nuclear dye. The percent cell death was calculated by the number of DEAD Green cells divided by the total number of NucBlue nuclear dye -positive cells within a well.

### Immunohistochemistry

Cells were fixed in 4% paraformaldehyde for 1 h, then permeabilized and blocked with 0.1% of Triton X-100 and 5 % donkey serum (Jackson ImmunoResearch Laboratories, West Grove, PA) to reduce nonspecific background. After an overnight incubation at 4°C with the primary antibodies [anti-*β*-tubulin III (StemCell Technology, 60100AD), anti-Map2a (Abcam, ab5392), and anti-Thy1 (Abcam, ab133350)], cells were washed with PBS and incubated with secondary AlexaFluor-conjugated antibodies appropriate for the species (Jackson ImmunoResearch Laboratories, 1:500).

### BacMam infection for live imaging

For fluorescent labeling of early endosome, late endosome, lysosomes, and synaptophysin, the cells were infected with CellLight™ Early Endosomes-GFP (C10586), CellLight™ Lysosomes-GFP (C10507), CellLight™ Late Endosomes-RFP (C10589), or CellLight™ Synaptophysin-RFP (C10610) (BacMam Technology, ThermoFisher Scientific, Carlsbad, CA), respectively, according to the supplier’s instructions 48 h before bacterial infection. For live imaging NucBlue nuclear dye was added before image acquisition. For live imaging of *P. gingivalis* internalization, the NeuO neuron-specific tracer (NeuroFluor™ NeuO, StemCell Technology, 01801) and the Lysotracker (LysoTracker™ Red DND-99, ThermoFisher, L7528) was added to the cultures 1 h before acquiring the images and 24 h after *P. gingivalis* infection.

### Image acquisition and analysis

Images were acquired with the ImageXpress Micro Confocal High-Content Imaging System (Molecular Devices, Sunnyvale, CA) with a 40x objective. Series of images were acquired at different planes along the focal axis (Z-stack). Stacks from 7 up to 75 planes separated by 2 or 0.2*μ*m, respectively, were acquired, covering approximately 15*μ*m in depth. For 3D rendering, all individual images were saved; for 2D analysis, 2D maximum projection images were saved. Automated, built-in MetaXpress Analysis Macros such as Count Nuclei, *Live/dead*, and *Cell scoring* were used to quantify NucBlue-positive cells, dead cell, and neuron numbers, respectively. For the quantification of synaptophysin positive boutons, *P. gingivalis* positive puncta, and counts of early endosome, late endosome, and lysosome, the MetaXpress custom module *Top Hat* followed by the *Count Round Objects* was utilized. Co-localization of *P. gingivalis* in the different cellular compartments was quantified with *Logical Operation* using the *AND* function. Percent *P. gingivalis* within a compartment was calculated by the number of co-localized *P. gingivalis* divided by the total number of *P. gingivalis.* The number of synaptophysin-positive boutons was standardized to the average counts of non-infected neurons at 24-h time point.

### Transmission electron microscopy

Neurons were grown on PLO and Laminin coated Permanox culture dishes (19340-72, Electron Microscopy Sciences, EMS, Hatfield, PA) for 3 weeks. 48 h after *P. gingivalis* infections, the samples were rinsed in PBS and fixed in 2.5% glutaraldehyde in PBS (EMS, 15980) overnight at 4°C. The next day, cells were post-fixed in 1% osmium tetroxide for 1 h at RT, rinsed in phosphate buffer (EMS, 19340-72), dehydrated in a graded ethanol series, and infiltrated with Poly/Bed^®^ epoxy resin (Poly/Bed^®^ 812 Mini Kit, 21959-1) according to the manufacturer’s instructions (Polysciences, Warrington, PA) using propylene oxide as a transition fluid. The samples were embedded in resin and polymerized for 24 h at 60°C. Ultrathin sections (70–80 nm) were cut using a diamond knife, collected on copper grids (200 mesh), and stained with 3% alcoholic uranyl acetate (6 min) and Reynold’s lead citrate (2 min). Grids were examined using the JEOL JEM-1400 transmission electron microscope. Representative digital images were obtained using the Gatan Orius SC1000 CCD Camera and DigitalMicrograph digital imaging software.

### Statistical analysis

All statistical analyses were done in GraphPad Prism. The analysis used for each data set is described in the accompanying figure legend. Data are expressed as mean±95% confidence interval or if presented in log scale as geometric mean±geometric standard deviation. Statistical analyses for significant differences were performed with Student’s *t*-test, two-way ANOVA (parametric data sets), or Kruskal-Wallis (non-parametric analysis) followed by Tukey’s posthoc test or Dunn’s multiple comparison where appropriate. The criterion for statistical significance was *p* < 0.05.

### Data availability

The datasets used and/or analyzed during the current study are available from the corresponding authors on reasonable request.

## RESULTS

### Neurotoxicity of P. gingivalis infection in neuron cultures is concentration and time dependent

**Fig.1:**
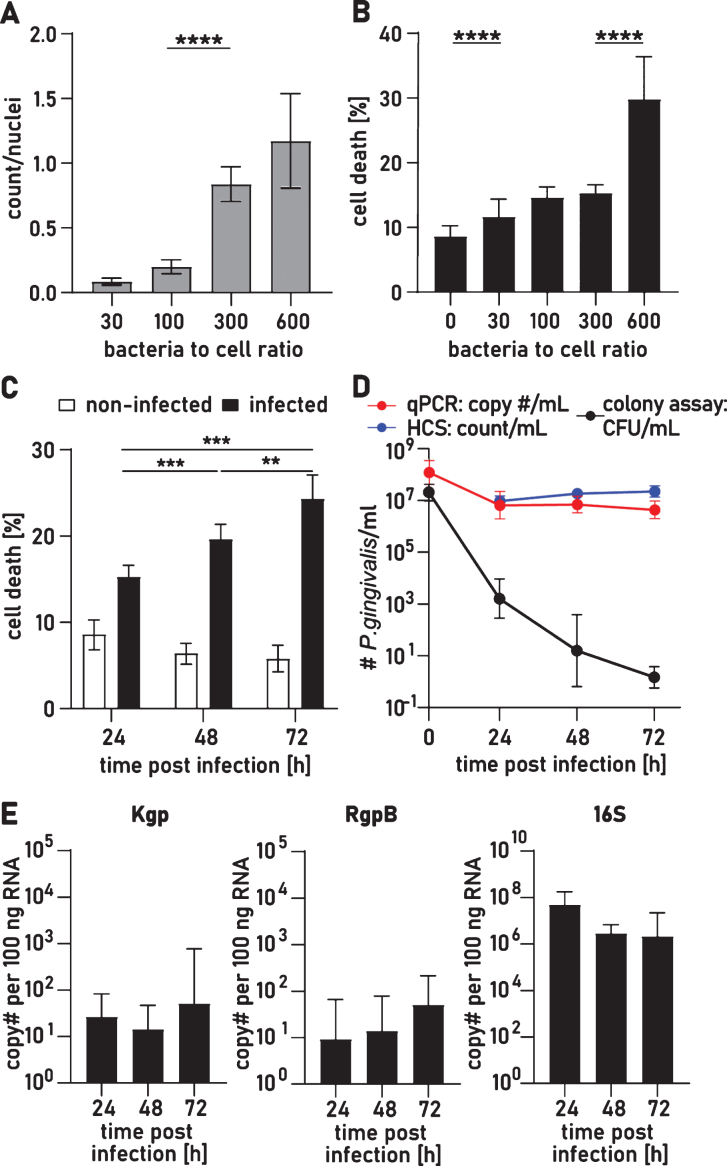
Viability of iPSC derived neurons and *P. gingivalis* for up to 72 h after infection. A) The number of *P. gingivalis* colocalized with neurons increases with increased numbers of *P. gingivalis* added to cultures. Kruskal-Wallis test with Dunn’s multiple comparison test: ****p < 0.001. B) Neuron cell death assay quantified by the ratio of dead cells over the total number of nuclei 24 h after infection with different BCRs. Kruskal-Wallis test with Dunn’s multiple comparison test: ****p < 0.0001. C) Neuron cell death assay 24 h, 48 h, and 72 h after infection with BCR 300. Two-way ANOVA with Tukey’s multiple comparison test: **p < 0.01, ***p < 0.001. D) Quantification of bacteria 24 h, 48 h, and 72 h after infection with BCR 300 by colony assay (CFU), qPCR, and high content screening (HCS). E) RT-qPCR analysis of neuron lysates for Kgp, Rgp mRNA, and *P. gingivalis* specific 16 S rRNA. A-C) Mean with 95% confidence intervals. D-E) Geometric mean with geometric standard deviation.

To determine the ability of *P. gingivalis* to invade and persist in neuron cultures, we cocultured *P. gingivalis* and neurons in a low oxygen incubator (5% O2, 5% CO2) for 24 h. The partial oxygen pressure in the brain has been reported as 20–25 mmHg [27] and culture conditions at 1–5% oxygen model the physiological level for neurons which is beneficial for long term cultures [28]. *P. gingivalis*, an obligate anaerobe, has been shown to tolerate up to 6% O2 in reduced hemin cultures [29] enabling a neuron infection model in oxygen conditions suitable for both pathogen and host cells. To optimize bacteria to cell ratio (BCR) for a prolonged neuron infection model, we titrated the BCR to determine the best infection rate with highest neuronal viability for up to 72 h. Neurons were infected with a range of 30 to 600 BCR. Bacteria that were not attached, tightly associated with, or internalized in the neurons were washed off and the number of *P. gingivalis* per nucleus after 24 h of co-culture were quantified using confocal high content screening (HCS) (Fig. 1A). A dose response to infection was seen with higher BCR producing higher load of *P. gingivalis* infection and the most significant increase between 100 and 300 BCR.

After 24 h, *P. gingivalis* induced cytotoxicity was significantly higher for BCR 30, 100, and 300 with averages of 11.7±5.4%, 14.6±3.4%, and 15.3±5.5% dead cells, respectively, compared to 8.6±6.0% in control cultures (Fig. 1B) but with no dose dependent increase. Infection with BCR 600, however, significantly increased the number of dead cells to 29.8±5.3%. Since BCR 300 resulted in the highest infection rate with low acute cytotoxicity, we quantified cell death at 48 h and 72 h after infection with BCR 300 (Fig. 1C). There was a significant time dependent increase in cell death to 19.7±4.7% at 48 h and to 24.35±7.6% at 72 h. 75% of neurons survived for at least 72 h of infection allowing these cultures to be utilized for further investigation of mechanistic effects of *P. gingivalis* and its proteolytic gingipains on human neurons.

### Infection of neurons remains stable for up to 72 h and P. gingivalis enters a viable but non- culturable state

To determine the ongoing persistence of infection, we removed nonattached bacteria with PBS washes and, at multiple timepoints, quantified the remaining attached and intraneuronal bacteria by confocal HCS of the cultures followed by qPCR analysis of the neuron cell lysates. The HCS and qPCR analysis showed comparable neuron infection of *P. gingivalis* which remained stable from 24 h to 72 h (Fig. 1D). A colony forming unit assay (CFU assay) found that the infection rate at 24 h was 0.1%, and CFU numbers dropped another 100-fold from 24 h to 48 h (Fig. 1D). After 72 h of infection, very few cultures could be grown from cell lysates. Since neuron associated *P. gingivalis* persisted at such a high level, we surmised that after infecting neurons *P. gingivalis* might enter a viable but not culturable (VBNC) state. *P. gingivalis* displaying VBNC has been previously reported and VBNC is a common adaptation for intracellular pathogens to persist in a hostile environment [21, 30, 31]. To demonstrate viability of non-culturable *P. gingivalis*, we performed RT-qPCR analysis to detect active transcription of *P. gingivalis* specific genes to mRNA. RNA is quickly degraded after transcription and the presence of specific RNA has been described as a gold standard method to investigate VBNC [32, 33]. RNA was isolated from infected neuron cultures at 24 h, 48 h, and 72 h and mRNA was detected and quantified for Lysine gingipain (Kgp), arginine gingipain (RgpB) as well as *P. gingivalis* specific 16 S rRNA at all time points (Fig. 1E).

### P. gingivalis aggregates are engulfed in neuronal membrane and express Rgp and Kgp

**Fig.2:**
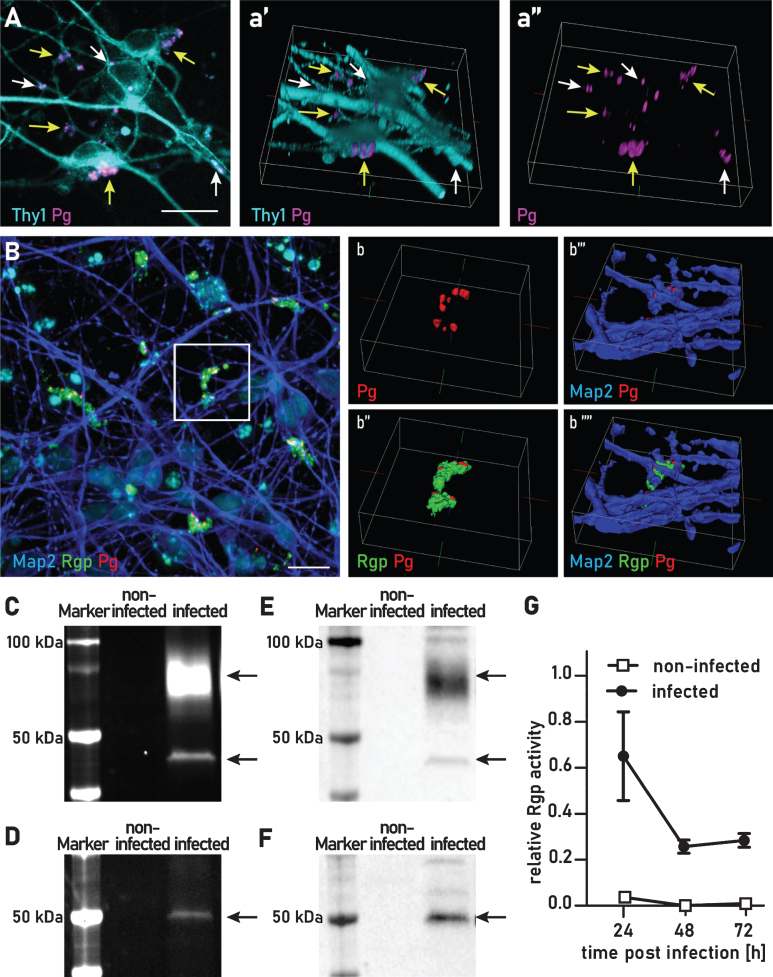
*P. gingivalis* is internalized and secretes active gingipains for up to 72 h. A) Maximal projection of a confocal image stack of *P. gingivalis* (magenta) infected neurons stained for the membrane specific protein Thy1 (cyan). a’) 3D reconstruction. a”) Same as a’ without the Thy1 channel. Yellow arrows point to aggregates of *P. gingivalis* partially engulfed in membrane. White arrows point at *P. gingivalis* completely internalized in the neuronal membrane. Scale bar 20*μ*m. B) Maximal projection of confocal image stack immunofluorescence stained for Rgp (green), *P. gingivalis* (red), and MAP2 (blue): secreted Rgp creates a field around neuron associated bacterial clusters. b-b””) 3D iso-surface reconstruction of the area indicated in B. b) MAP2 and Rgp channels omitted. b”) MAP2 channel omitted. b”’) Rgp channel omitted. b””) All channels visible. Gel electrophoresis of cell lysates incubated with a Cy-5 conjugated activity probe for Rgp (C) and Kgp (D). Arrows indicate the expected protein sizes at 45 kDa and 70 kDa for Rgp (C) and 50 kDa for Kgp (D). E and F are the corresponding western blots to the gels depicted in C and D, respectively. Blots were stained with antibodies against Rgp (E) and Kgp (F). Full length gels and western blots are presented in Supplementary Figure 5. G) Colorimetric activity assay for Rgp activity from cell lysates at 24 h, 48 h, and 72 h after infection.

3D reconstructions from multichannel confocal image stacks were used to investigate if *P. gingivalis* was internalized into neurons in culture 24 and 48 h after infection, *P. gingivalis* was labeled with a fluorescence marker covalently bound to the bacterial membrane, and neurons were stained by immunofluorescence with the neuron-specific membrane protein marker Thy1/CD90. (Fig. 2A). Various sized aggregates of *P. gingivalis* appeared to reside in a membrane swelling on neurites and cell bodies. However, some of the aggregates appeared to be only partially covered by Thy1 positive membrane. Immunostaining with microtubule associate protein 2 (MAP2) showed MAP2 positive particles in and around the bacterial aggregate, supporting the finding that these aggregates are surrounded by neuronal cell products (Fig. 2B). *P. gingivalis* survival in the host cell has been shown to depend on the activity of Kgp and Rgp [1, 24–26]. Consequently, we investigated whether Kgp and Rgp were present in infected neuron cultures. Immunofluorescence staining with CAB101, an antibody raised against Rgp [5], formed a large diffuse field (green) around intraneuronal *P. gingivalis* (red) 48 h after infection suggesting consistent gingipain secretion of gingipains and confirming transcriptional activity of gingipain described above (Fig. 2B).

### Gingipains in infected neuron cultures are proteolytically active for at least 72 h after infection

To determine if Kgp and Rgp retain active protease activity, we incubated the infected neuron cultures with Cy5-conjugated activity probes specific for Rgp and Kgp (COR619 and COR553, respectively). These probes are designed to be highly specific and to irreversibly bind to the active sites of Rgp and Kgp. Only active Rgp or Kgp is detected with this method. To probe intracellular and tightly cell-associated gingipains, cultures were washed with PBS before lysing. Active gingipains were then detected with the addition of the gingipain activity probes. At 24 h after infection, we were able to detect Rgp activity as measured by fluorescent probe detection of Rgp at the predicted molecular weights of 45 kDa and at 70 kDa (Fig. 2C). In the complementary experiment, we were able to detect active Kgp with a single band of 50 kDa (Fig. 2D). As a further control for the identity of Rgp and Kgp detected with the activity probes the identity of these bands was confirmed by immunoblotting with CAB101 and CAB102, polyclonal antibodies raised against Rgp and Kgp, respectively (Fig. 2E, F).

By using a highly sensitive substrate-based enzyme activity assay we were able to quantify Rgp activity of neuron internalized *P. gingivalis* for at least 72 h. We found a slight drop of activity when comparing 24 h of infection with 48 h, but this activity remained stable from 48 h to 72 h (Fig. 2G). We also assayed for Kgp activity, however Kgp activity was below the detection level of this Kgp substrate-based assay. Thus, in neuron cultures infected with *P. gingivalis* proteolytically active Kgp and Rgp are actively produced for at least 72 h after infection.

### P. gingivalis interacts with the neuron membrane and is internalized

**Fig.3:**
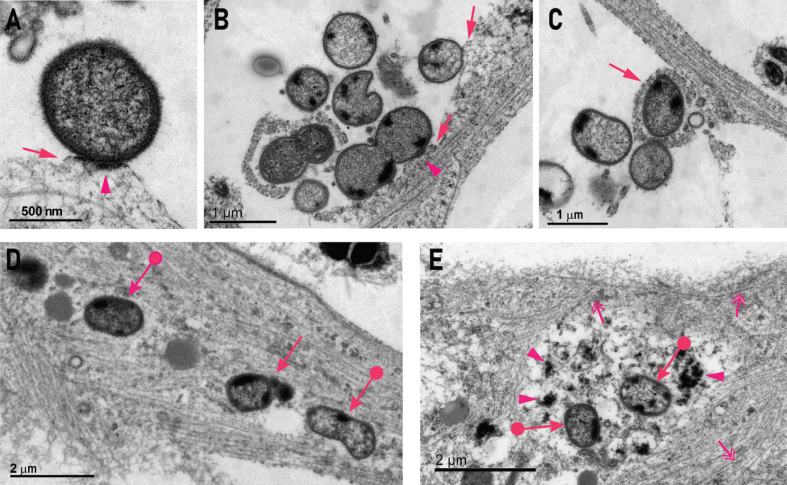
*P. gingivalis* attaches to neurons, is internalized, and is associated with disruption of the cytoskeleton. A,B) Examples of extracellular *P. gingivalis* contacting a neurite and inducing a membrane swelling resembling an actin pedestal (arrow). This was associated with an electron dense band along the contact surface to *P. gingivalis* (arrowhead). C) Some bacteria were internalized by membrane swellings on neurites (arrow). D) An example of two *P. gingivalis* organisms (circle arrows) residing within a neurite without being bound by a distinct surrounding membrane and one *P. gingivalis* inside a membrane (arrow). E) Two *P. gingivalis* within the cell body that are non-membrane bound (circle arrows) and are surrounded by heterogenous electron dense matrix (arrowheads) with a peripheral zone of neurofilaments and microtubules (double headed arrows).

Transmission electron microscopy (TEM) was used to investigate internalized and attached *P. gingivalis* at the ultrastructural level. In the TEM images, healthy bacteria could be identified by a round structure, an electron dense double cell membrane and visible fimbriae (Fig. 3A-C). Extracellular bacteria could be seen contacting the neurite membrane and an electron dense band resembling a pedestal was occasionally visible. Such pedestals have also been described in scanning electron microscopy studies on *P. gingivalis* infected coronary artery endothelial cells and human umbilical vein endothelial cells [23, 34]. Pedestal formation is induced by pathogen-host interactions that are highly specific and not well understood for *P. gingivalis*, but commonly involve the rearrangement of the actin cytoskeleton and result in the pathogen entering the host cell [20, 35]. Some bacteria appeared to be in the process of being endocytosed into the cell (Fig. 3B, C). None of the bacteria observed during endocytosis were enclosed in an endocytic membrane (Fig. 3C, D), resulting in non-compartmentalized, free bacteria in the cytoplasm. Neurofilaments and microtubules around free bacteria were disrupted and the bacteria were surrounded by electron dense cell debris (Fig. 3E).

### Over time intraneuronal P. gingivalis increasingly reside in lysosomes

**Fig.4:**
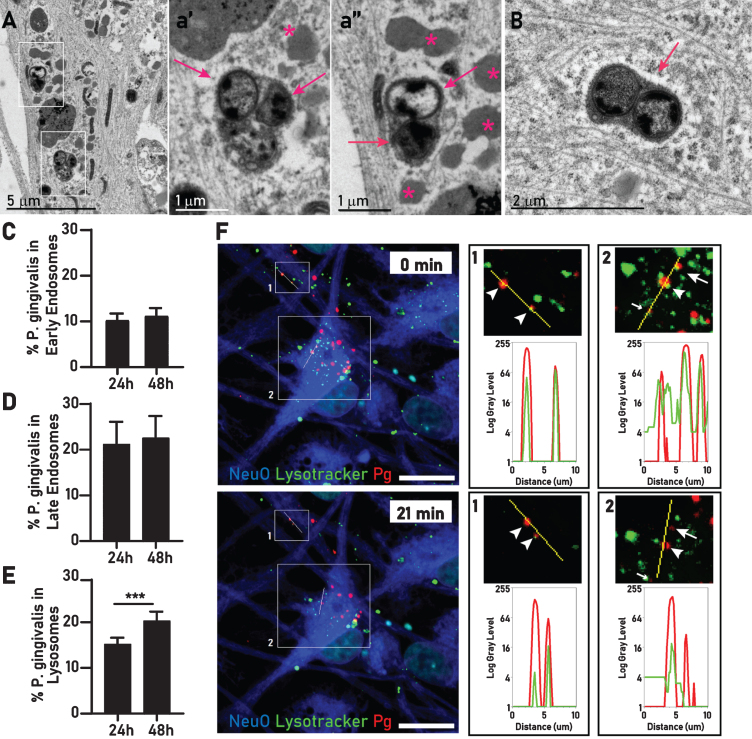
Intraneuronal *P. gingivalis* is found in the endosomal/lysosomal pathway. A) Two groups of *P. gingivalis* (white rectangles) within membrane bound structures resembling lysosomes. a’) Enlarged inset of A (lower square) showing an aggregate of two intact bacteria (arrows) and a partially degraded bacterium a”) Enlarged inset of A (upper square) a pair of intact bacteria (arrows) surrounded by an accumulation of lipid droplets (stars). B) Two *P. gingivalis* inside a single membrane (arrow), the surrounding cytosol contains rough endoplasmic reticulum and neurofilament looks unperturbed except for a small electron lucent halo surrounding the bacteria containing compartment. Percentage of internalized *P. gingivalis* colocalizing with early endosomes (C), late endosomes (D), and lysosomes (E). Mean with 95% confidence interval. Student’s *t*-test, ***p < 0.005. F) Live imaging of *P. gingivalis* (red), lysotracker (green), and NeuO (blue). Line scans in a neurite (inset 1) and the cell body (inset 2) confirm colocalization of *P. gingivalis* with migrating lysosomes over time. In inset 1, both bacteria continue to colocalize with lysotracker (arrowheads). In inset 2, three bacteria colocalize at time 0. At 21 min, one remains in the lysosome (arrowhead), one has moved away from the line scan (small arrow), and one is not colocalizing with lysotracker anymore (arrow).

Much of the intracellular bacteria were located inside a single membrane compartment resembling a lysosome. These bacteria appeared to be healthy or in various stages of disintegration (Fig. 4A, B). Commonly, around compartmentalized bacteria we observed an electron light halo surrounding the membrane with the adjacent cytoplasm intact (Fig. 4B). To further characterize the lysosome-like structure observed in TEM, we expressed early endosome, late endosome, and lysosome specific markers (Rab5, Rab7, and Lamp1, respectively) in neurons using a baculovirus-based transduction system (BacMam) and quantified the colocalization with *P. gingivalis* by HCS at 24 h and 48 h after infection. The percentage of *P. gingivalis* in early endosomes and in late endosomes remained stable between 24 h and 48 h (Fig. 4C-E). However, the percentage of *P. gingivalis* colocalizing with lysosomes increased by 33% at 48 h compared to 24 h (Fig. 4E), suggesting that over time *P. gingivalis* may accumulate in these organelles. Using lysotracker staining in neurons infected with fluorescently labeled *P. gingivalis* we observed the colocalization of *P. gingivalis* and lysosomes over time and colocalization of *P. gingivalis* with lysotracker could be confirmed for bacteria moving in neurites and in cell bodies (Fig. 4F, Supplementary video).

### P. gingivalis infection induces multivesicular bodies and dystrophic neurites containing autophagic vacuoles and secondary lysosomes

**Fig.5:**
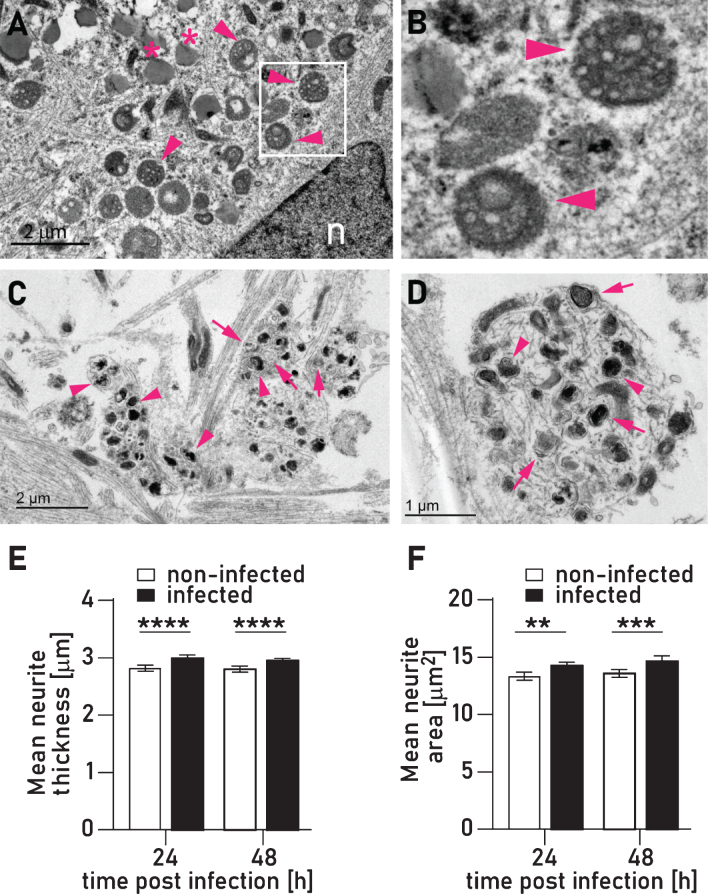
Infection with *P. gingivalis* causes an accumulation of autophagosomes and multivesicular bodies. A) Accumulation of multivesicular bodies (arrow heads) in the perikaryon of neuron infected with *P. gingivalis*. B) Magnified inset of A. C,D) Two examples of variably sized membrane bound vesicles containing heterogeneous amorphous electron dense material interpreted as secondary lysosomes (arrowheads) or occasional concentric lamellar bodies interpreted as autophagosomes (arrows). Quantification of mean neurite thickness (E) and mean neurite area (F) showed significant increases in infected neuron cultures. Mean with 95% confidence interval; **p < 0.01, ***p < 0.001, ****p < 0.0001.

Neuron cultures infected with *P. gingivalis* displayed cytopathological features in areas where no discernable bacteria were observed. In some neurons, multivesicular bodies and lipid droplets accumulated in the perikaryon (Fig. 5 A, B). In noninfected neuron cultures, only very few multivesicular bodies were present; however, we did find lipid droplets close to lysosomes (Supplementary Figures 2 and 3). Additional TEM images of noninfected neurons are presented in Supplementary Figures 1-4.

In addition to the pedestals induced by direct interaction with *P. gingivalis* described above, our TEM analysis revealed an additional type of membrane swelling where the membrane appeared to be intact and was filled with variably sized (less than 1*μ*m diameter) membrane bound vesicles containing heterogeneous amorphous electron dense material we have interpreted as secondary lysosomes or concentric lamellar bodies interpreted as autophagosomes (Fig. 5C, D). These structures very closely resemble autophagic vacuoles that form in dystrophic neurites in the brains of AD patients [36, 37]. By using image analysis, we were able to quantify and confirm that mean neurite thickness and area is increased in *P. gingivalis* infected neurons (Fig. 5E, F).

### Tau protein is degraded in P. gingivalis-infected neurons and phosphorylated over time

**Fig.6:**
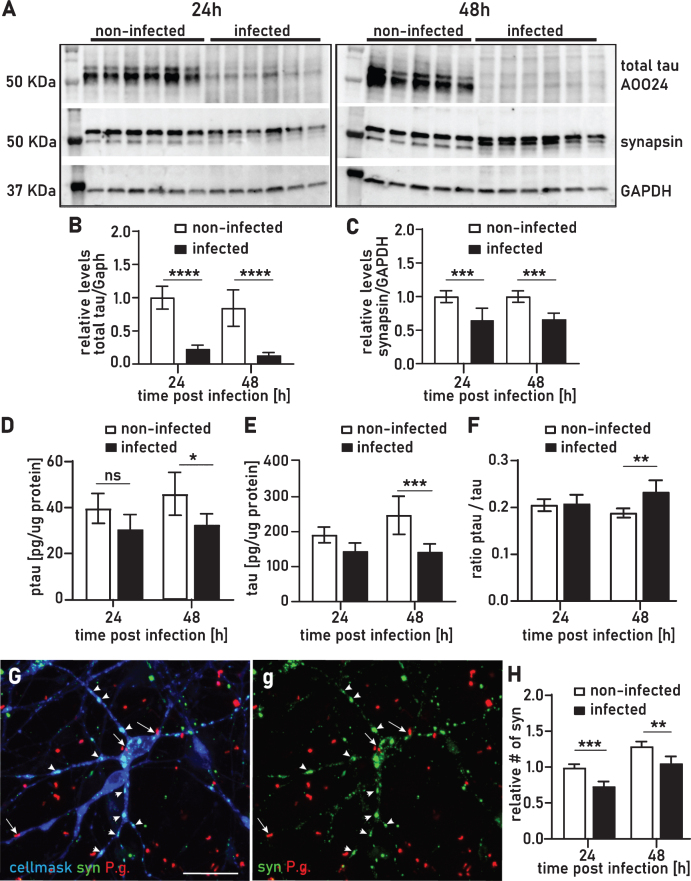
*P. gingivalis* degrades tau, increases the ratio of ptau/tau and decreases synapse density. A) Western blots stained with total tau and synapsin antibodies for infected and noninfected lysates at 24 h and 48 h after infection. GAPDH was included as a loading control. Full length western blots are presented in Supplementary Figure 6. B,C) Densitometry of western blots depicted in A normalized to GAPDH. Mean with 95% confidence interval, 1-way ANOVA with Tukey’s multiple comparison test ****p < 0.0001, ***p < 0.001. Multiplex ELISA quantification of ptau(T231) (D), total tau (E), and the calculated ptau(T231)/total tau ratio (F). Mean with 95% confidence interval, 1-way ANOVA with Tukey’s multiple comparison test: *p < 0.05, **p < 0.01, ***p < 0.001. G) Multichannel maximum projection of a confocal stack with synaptophysin (green), *P. gingivalis* (red), and a universal membrane marker (blue) used as a cell mask for high content screening. The blue channel has been omitted in the right-side panel (g). Arrows point at *P. gingivalis* associated with neurons; arrow heads point at synaptophysin positive synaptic buttons. Scale bar 50*μ*m. H) Quantification of synaptic buttons 24 h and 48 h after infection. Mean with 95% confidence interval, 2-way ANOVA with Dunnett’s multiple comparison test: **p < 0.01, ***p < 0.005.

We have previously shown that recombinant tau-441 protein and high molecular weight tau in SH-SY5Y cells is highly susceptible to protease fragmentation by gingipains [1]. Western blot analysis of lysates from 24 h and 48 h infected neurons showed strong degradation of tau compared to non-infected controls (Fig. 6A, 6B) which confirms our previous findings. One of the hallmarks of the AD brain is an abnormal hyperphosphorylation of tau [38–40] and we investigated whether tau hyperphosphorylation might be a direct consequence of *P. gingivalis* infection. We performed western blot analysis for ptau(S396), a site that has been reported to be phosphorylated early in the onset of AD [38–40] and found that in infected cultures ptau was comparably degraded to the tau blot (Supplementary Figure 7). Because in the western blot analysis tau was fragmented to a degree that the quantitation of ptau/total tau was not feasible, we used a multiplex ELISA assay to quantify both total tau and ptau (T231), a phosphorylation site identified in AD brains [41, 42] to determine potential effects on hyperphosphorylation. Interestingly, ptau/tau ratio was increased by 23% after 48 h but not after 24 h (Fig. 6F). Both tau and ptau were equally reduced at 24 h but at 48 h tau was comparably more reduced than ptau (T231) (Fig. 6D, 6E).

### P. gingivalis neuronal infection results in synapse loss

High content screening analysis with immunofluorescence staining for synaptophysin revealed a 26% and 19% decrease in synapse density on neurons at 24 h and 48 h of infection, respectively (Fig. 6G, 6H). This finding was corroborated by a comparable decrease in synapsin detected by western blot (Fig. 6C). Synapse loss is one of the earliest pathological events in the onset of AD and both synaptophysin and synapsin have been reported to be decreased in vulnerable areas of AD affected brains [43].

## DISCUSSION

This study demonstrates the infection of neurons with *P. gingivalis in vitro*. Intraneuronal *P. gingivalis* was detected within single membrane, lysosome-like structures or unbound in the cytoplasm. Confocal high content screening found colocalization with endosome markers Rab5 and Rab7 as well as the lysosome markers Lamp1 and lysotracker. In addition, we demonstrate that after 72 h of infection, *P. gingivalis* is in a viable but non-culturable state and expresses both active Rgp and Kgp. Larger aggregates of bacteria appeared partially engulfed by neuronal membrane and produced significant amounts of gingipain detectable by immunohistochemistry. Given the cluster formation and apparent coating with gingipains, we hypothesize these aggregates might represent biofilm covered repositories of *P. gingivalis* [44]. Consequently, comparable to periodontal *P. gingivalis* colonies, these aggregates appear to be producing gingipains and bacteria and continuously infect neurons over longer time periods.

In the ultrastructural analysis, the cytoskeleton surrounding cytosolic *P. gingivalis* appeared to be disintegrating and analysis of the cell lysates revealed significant degradation of tau protein. We have previously shown the proteolytic activity of gingipains on tau protein and that fragmentation of tau after *P. gingivalis* infection of SH-SY5Y cells was gingipain dependent [1] and active gingipains were demonstrated in infected neurons in the current study.

Intriguingly, the ratio of phosphorylated tau increased only 48 h after infection but was unaffected at 24 h, even though total tau was decreased at both times. This suggests that either hyperphosphorylated tau has greater stability and might be a protective mechanism against gingipain mediated protein digestion and/or phosphorylation of tau is increased after prolonged infection with *P. gingivalis*. It has previously been shown that phosphorylation of tau protects tau against proteolytic degradation [45]. This result is consistent with a recent study showing that oral infection with *P. gingivalis* in wild type mice resulted in gingipains being identified inside neurons along with hyperphosphorylated tau that was not seen in uninfected mice [2].

It is generally accepted that one of the main functions of tau is the stabilization of microtubules [46–48]. Degradation of tau by gingipains and resulting hyperphosphorylation may result in destabilization or dysfunction of microtubule structures. Microtubules are crucial for axonal transport mechanisms such as vesicle or lysosome transport [49], and the maintenance of synapses depends on microtubule-based transport of synapsin and synaptophysin [50]. In our neuron cultures, we observed a significant loss of synapse density after infection with *P. gingivalis*. This was normalized to the MAP2 positive area or total protein and therefore is independent of neurotoxicity induced by infection. Synapse loss and decreased synapsin is one of the first neuropathological events in the onset of AD related dementia [43]. Synapse loss in AD has been attributed to impaired axonal transport, and ultrastructural analysis in the AD brain found comparable neuronal abnormalities to iPSC neurons infected with *P. gingivalis*, including an accumulation of multivesicular bodies in the perikaryon and a buildup of autophagic vacuoles [36, 51, 52].

In our cultures, we found autophagic vacuoles only in infected cultures. This may represent a defense mechanism by infected cells to fight intracellular bacteria [53]; however, there is also experimental evidence that autophagic vacuoles accumulate when retrograde transport is inhibited [36, 51]. The resulting phenotype in the TEM analysis is remarkably comparable to autophagic vacuoles we observed in infected neurons.

Thus, the hypothesis that *P. gingivalis* infection results in impaired microtubule transport is supported by synaptic loss, decreased synapsin, multivesicular bodies accumulating in the perikaryon, and dystrophic neurites containing autophagic vacuoles. Importantly, these effects have all been described as early events in the development of AD pathology. Therefore, our initial characterization of *P. gingivalis* infected neurons suggests that this is a model to study early neuropathological events of AD-related dementia.

The primary aim of this study was to demonstrate the ability of *P. gingivalis* to invade and persist in neurons, and we optimized the BCR to our investigation time frame within the first 72 h of infection. Consequently, our study was performed with a BCR of 300, which is likely much higher than what a neuron in the brain might encounter and the resulting degenerative effects might be more pronounced and more toxic than what is encountered at physiological levels. However, if we extrapolate the neurodegenerative effects of this subacute infection to a progressive, repeated low level, chronic infection state, the resulting neuropathological effects appears very comparable to an AD brain.

In conclusion, *P. gingivalis* can infect neurons and persistently express gingipains. The neuropathological phenotype after 48 h of *P. gingivalis* infection includes autophagic vacuoles in neurites, an accumulation of multivesicular bodies in the perikaryon, degradation and phosphorylation of tau, and the loss of synapses. Comparable features of neuronal degeneration combined with presence of gingipains are detected in AD brains, and we propose that human iPSC-derived neurons infected with *P. gingivalis* are a novel *in vitro* model to investigate the neuropathological mechanisms and treatment of AD.

## Supplementary Material

Supplementary Figures

Supplementary Video Time lapse imaging of infected neurons demonstrating colocalization of *P. gingivalis* with lysotracker. *P. gingivalis* (red), lysotracker (green), and NeuO (blue). Outline 1 is presented as inset 1 and outline 2 as inset 2 in Figure 4F. The movie has been compiled from maximal projections of confocal image stacks imaged at 30-s intervals for 1 h.
