# Supplementary material for: Alzheimer’s Disease-Like Neurodegeneration in Porphyromonas gingivalis Infected Neurons with Persistent Expression of Active Gingipains
Source: J Alzheimers Dis. 2020 Jun 15;75(4):1361–76. doi: 10.3233/JAD-200393 (PMC7369049; doi:10.3233/JAD-200393)
Supplement: Supplementary Figures [file jad-75-jad200393-s001.pdf]

Supplementary Material

Alzheimer's Disease-Like Neurodegeneration in *Porphyromonas gingivalis* Infected Neurons with Persistent Expression of Active Gingipains

Supplementary video: Time lapse imaging of infected neurons demonstrating colocalization of *P. gingivalis* with lysotracker. *P. gingivalis* (red), lysotracker (green), and NeuO (blue). Outline 1 is presented as inset 1 and outline 2 as inset 2 in Figure 4F. The movie has been compiled from maximal projections of confocal image stacks imaged at 30-s intervals for 1 h.

Supplementary Figures 1-4. TEM images of non-infected iPSC derived neurons after 48 h in culture.

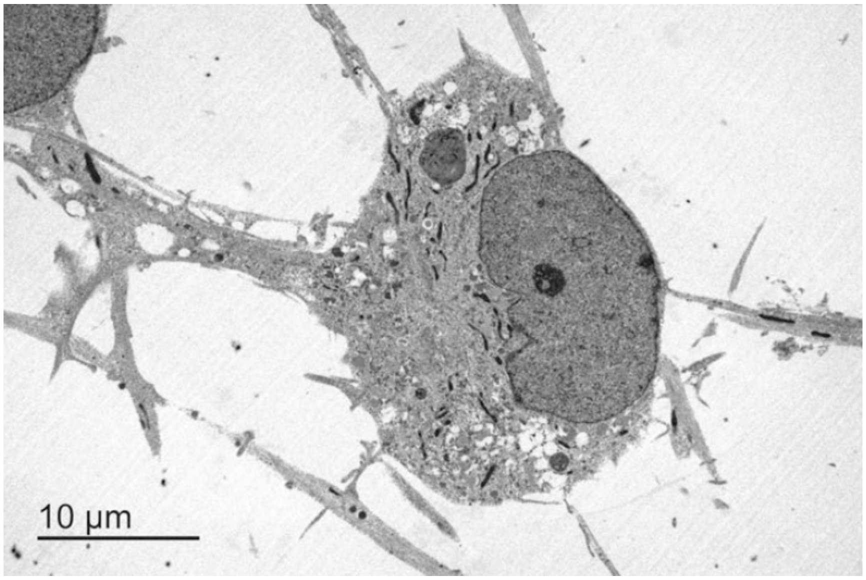

Supplementary Figure 1. Low magnification image contains one cell and a portion of a second cell nucleus. The nucleus is peripherally located and contains a single nucleolus.

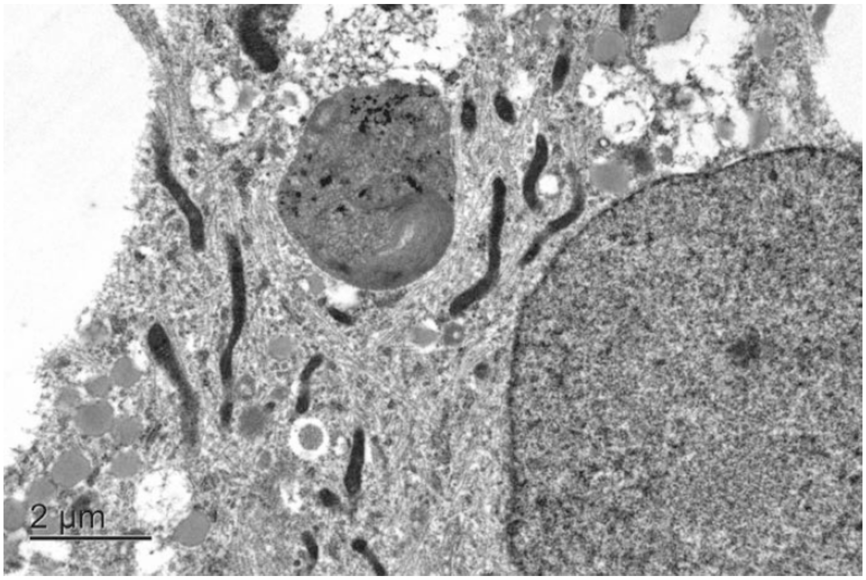

Supplementary Figure 2. The perikaryon contains multiple non-membrane bound electron lucent homogeneous lipid vacuoles, streams of intermediate filaments, elongated mitochondria, ribosomes, and a single approximately 3 µm diameter membrane bound lysosome containing heterogeneous electron dense matrix and concentric membranous whorls.

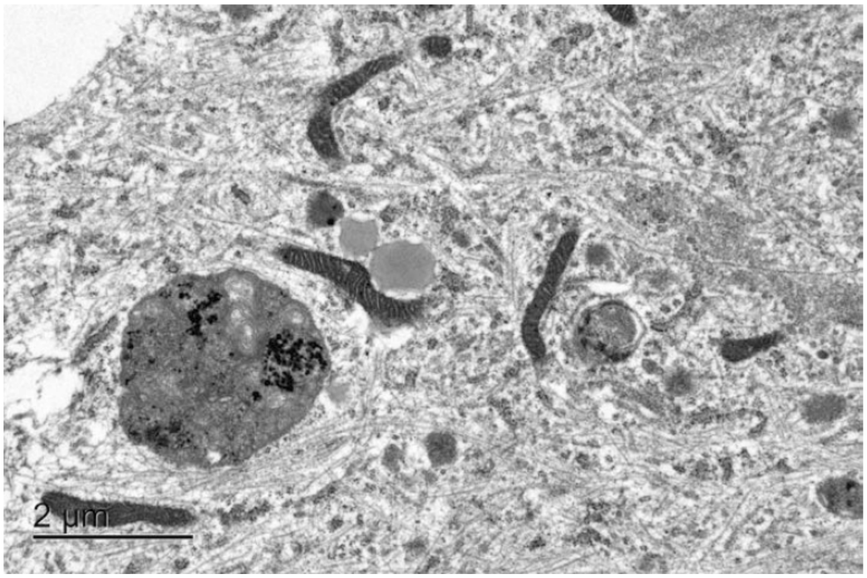

Supplementary Figure 3. The cytosol contains interlacing intermediate filaments, elongated mitochondria, ribosomes, rough endoplasmic reticulum, lipid vacuoles as previously described, and approximately 1.5-3 µm diameter organelles containing concentric membranous whorls and heterogeneous electron dense matrix.

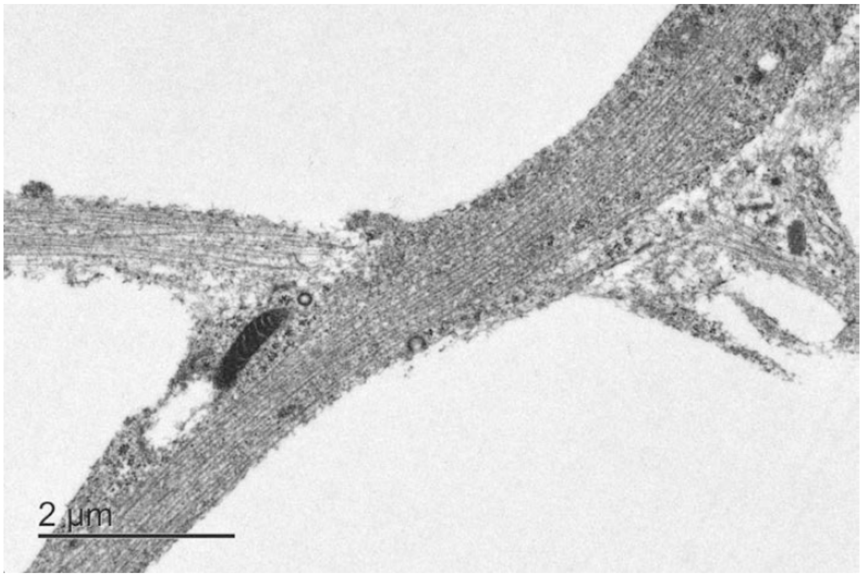

Supplementary Figure 4. Neurites contain intermediate filaments, mitochondria, and rough endoplasmic reticulum/ribosomes.

Supplementary Figure 5. Full length gels and western blots of Rgp and Kgp presented in Figure 2C-F.

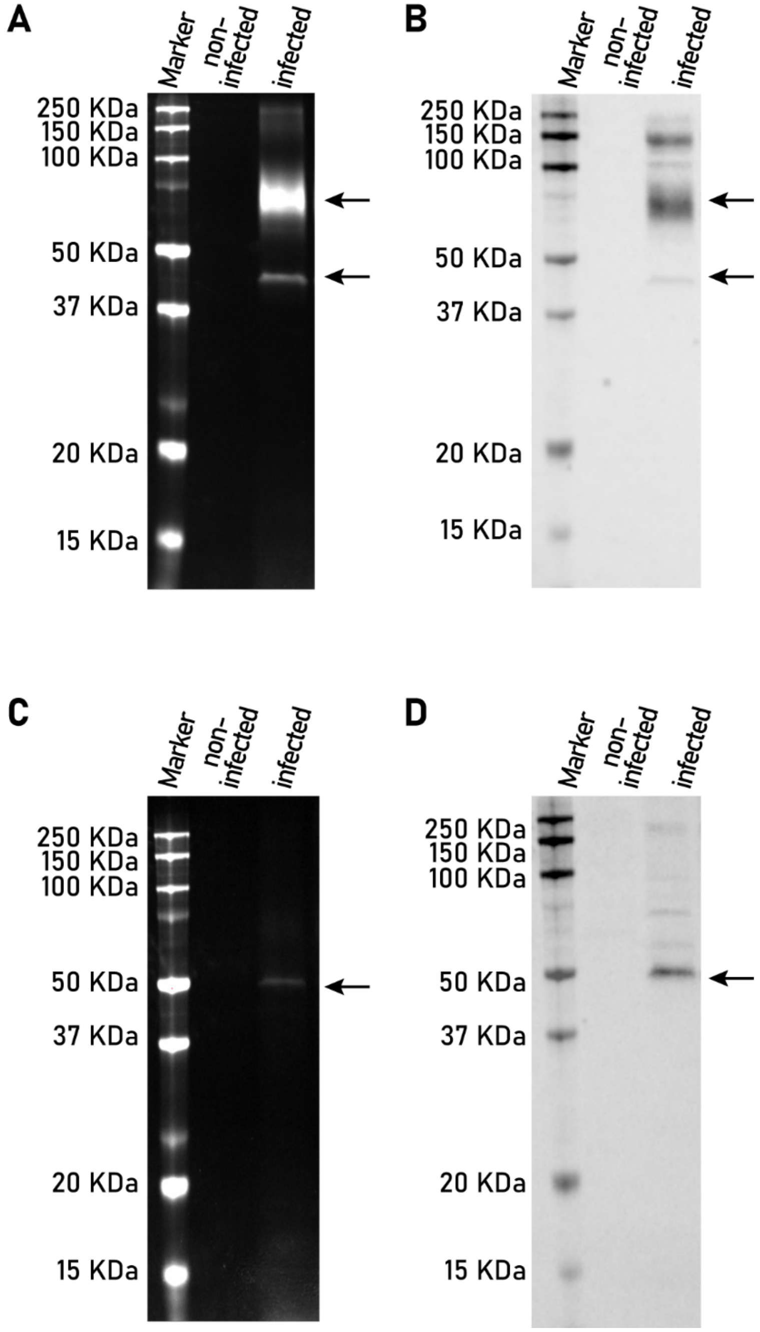

Supplementary Figure 5. Original full-length gels (A, C) and western blots (B, D presented in Fig. 5C, D and Fig. 5E, F, respectively). A, C) Gel electrophoresis of cell lysates incubated with a Cy-5 conjugated activity probe for Rgp (A) and Kgp (C). Arrows indicate the expected protein sizes at 45 kDa and 70 kDa for Rgp (A) and 50 kDa for Kgp (C). B and D are the corresponding western blots to the gels depicted in A and C, respectively. Blots were stained with antibodies against Rgp (B) and Kgp (D).

Supplementary Figure 6. Full length western blots for total tau and synapsin1 presented in Figure 6A.

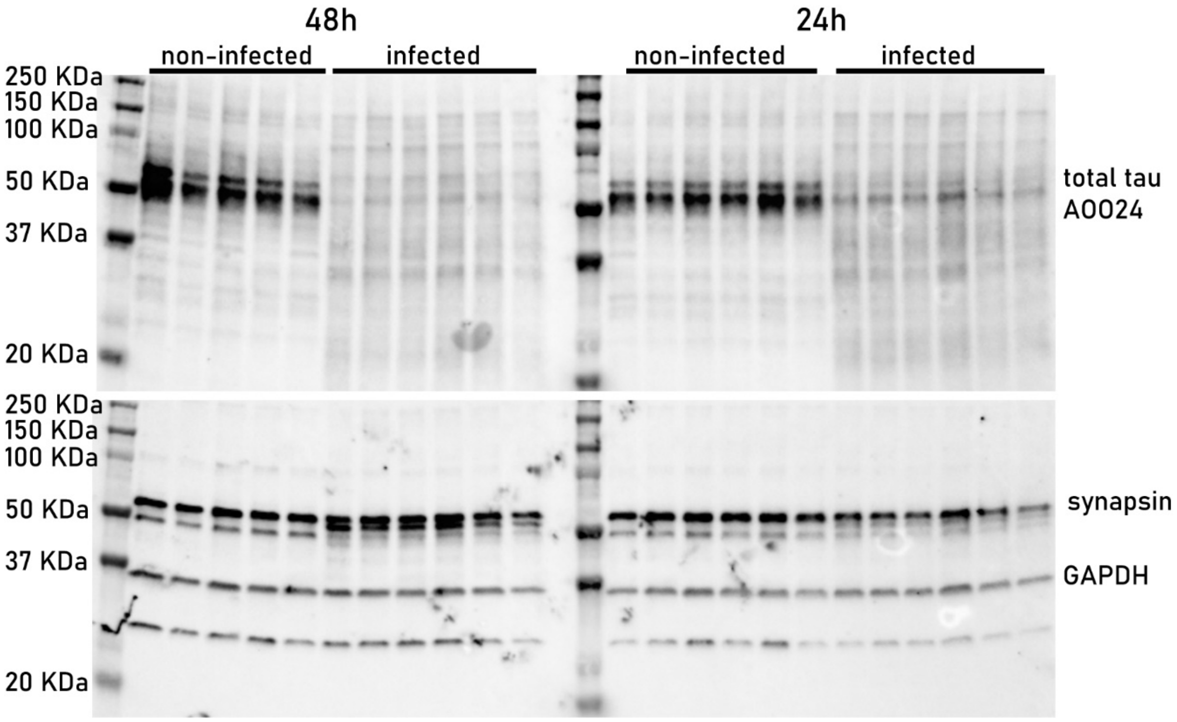

Supplementary Figure 6. Original full-length western blots presented in Figure 6A probed for tau (upper blot) and synapsin (lower blot).

Supplementary Figure 7. Western blot for ptau(S396)

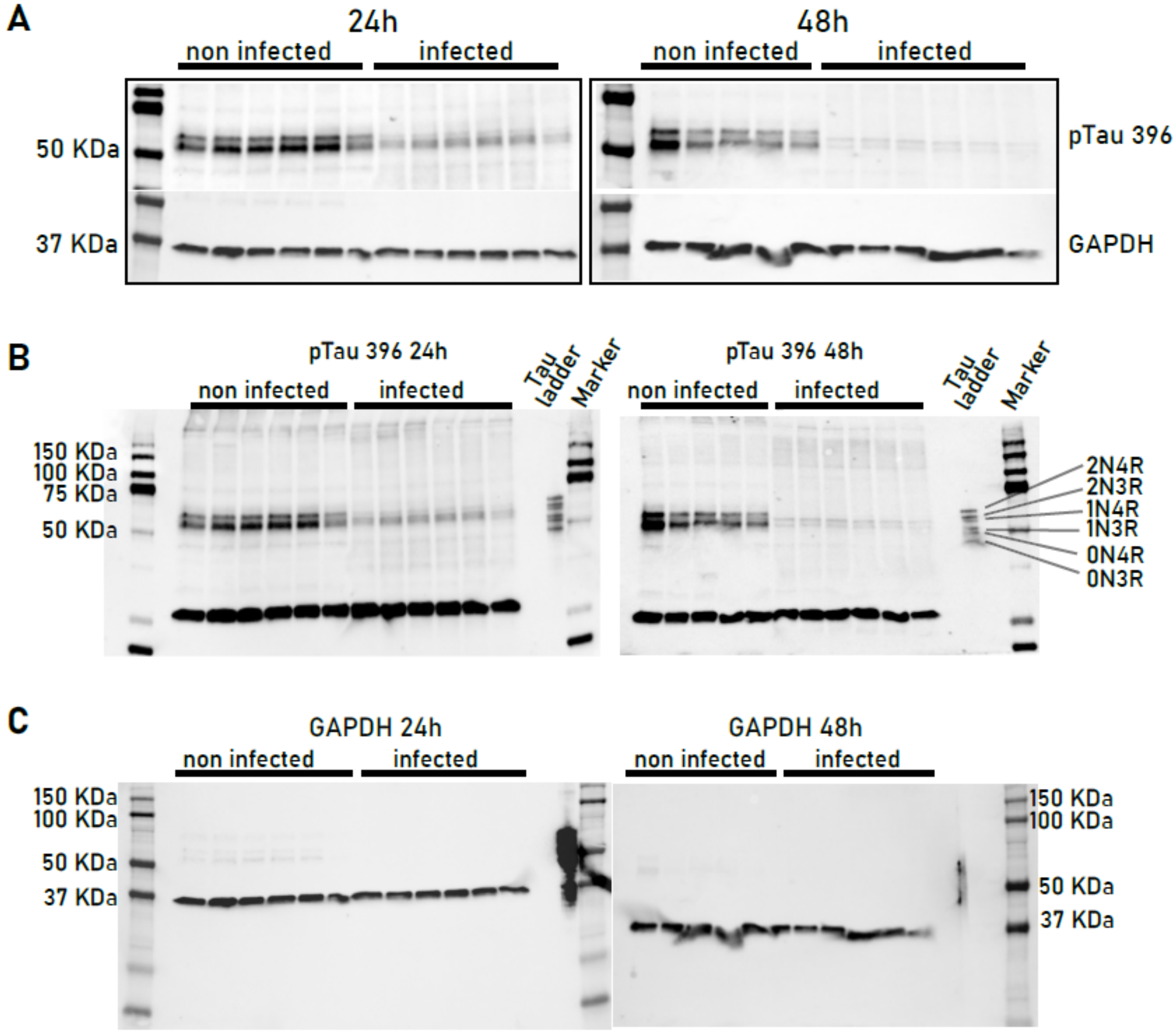

Supplementary Figure 7. Western blots for ptau(S396) 24 h and 48 h after infection. A) Cropped western blots for easy viewing of the relevant bands of ptau(S396) and GAPDH. B) Full size and non-cropped western blots for ptau(396). C) The blot from B was stripped and re-probed for GAPDH.
